# Comparison of Neurosensory Recovery of the Inferior Alveolar Nerve After Open and Closed Reduction for Mandibular Fractures: A Prospective Study

**DOI:** 10.7759/cureus.53175

**Published:** 2024-01-29

**Authors:** Naman Mundepi, Hibu Dora, Manish Sharma, Kshitija Patil, Ishita Rangnani, Sohini Saha

**Affiliations:** 1 Department of Oral and Maxillofacial Surgery, Kothiwal Dental College and Research Centre, Moradabad, IND; 2 Department of Orthodontics and Dentofacial Orthopaedics, Dora Dental Clinic, Papum Pare, IND; 3 Department of Oral Pathology, Jawahar Medical Foundation's ACPM (Annasaheb Chudaman Patil Memorial) Dental College, Dhule, IND; 4 Department of Oral and Maxillofacial Surgery, Jawahar Medical Foundation's ACPM (Annasaheb Chudaman Patil Memorial) Dental College, Dhule, IND; 5 Department of Surgery, Siksha 'O' Anusandhan University, Institute of Medical Sciences and SUM Hospital, Bhubaneswar, IND

**Keywords:** open reduction, closed reduction, recovery, inferior alveolar nerve, mandibular fracture

## Abstract

Introduction: Traumatic mandibular fractures are the most common fractures of the facial region and are associated with loss of neurosensation in the inferior alveolar nerve (IAN). The present study aimed to compare IAN recovery after traumatic mandibular fractures between the open and closed reduction methods.

Materials and methods: The study included 90 patients with traumatic mandibular fractures of the body, angle, and symphysis, divided into two groups of 45 patients: group 1 was treated with closed reduction and fixation with rich arch-bar fixation under local anesthesia, and group 2 was treated with open reduction and rigid internal fixation with 2-mm titanium mini plates and monocortical screws (6 mm), and the plate was fixed to the fractured bony fragments. All patients underwent neurosensory testing using the Zuniga and Essick algorithm at baseline (preoperative), one week after surgery (postoperative), at three months, and at six months of follow-up.

Results: No statistically significant differences were observed in IAN recovery between the groups. The most common site of fracture was the body (44% in group 1 and 56% in group 2). The maximum recovery was observed in the younger age group (25-30 years). At baseline, functional nerve recovery was observed in 40 cases (88%) in group 1 and 38 cases (84%) in group 2, and the difference was not statistically significant. Levels A and B tests were affected by surgical management and improved after three months. The total recovery in group 1 ranged from 60% to 80%, and that in group 2 ranged from 56% to 72%.

Conclusion: Based on the findings of the current study, both methods are recommended for surgical management of traumatic mandibular fractures with IAN recovery in 60-80% of cases six months postoperatively.

## Introduction

The mandible is acknowledged as the primary recipient of force during traumatic events, rendering it the most prevalent type of fracture due to its enduring nature resulting from such occurrences. Fractures of the mandible often lead to impairment of the inferior alveolar nerve (IAN) and modification of neurosensory capability. This could be attributed to the initial damage occurring when the IAN is situated within the fracture line, or it could be subsequent harm caused by the manipulation and stabilization of the fracture [1]. The occurrence of inferior alveolar nerve damage (IAND) following the treatment of fractures varies between 0.4% and 91.3%. Assessments of enduring IAN neurosensory impairments following mandibular fractures fall within the range of 0.9%-66.7% [2]. The wide array of prevalence estimates serves as evidence for the absence of consistency in both recorded occurrences and methodologies employed to evaluate deficiencies in neurosensory function [3].

In the clinical setting, numerous approaches exist for neural assessment, such as imaging techniques (magnetic resonance imaging, computed tomography, positron emission tomography), electrodiagnostic tests (nerve conduction velocity test, electroencephalogram, electromyography), and functional imaging (functional magnetic resonance imaging, near-infrared spectroscopy) [3]. These methods range from simply asking patients for their subjective experience of any neurosensory impairment to employing complex clinical neurosensory testing (CNT). Subjective assessments tend to be unreliable as they may yield diverse neurosensory deficiencies compared to objective evaluations [4]. In 1992, Zuniga and Essick proposed a simple algorithm to apply at the chair side for neurosensory evaluation [5].

There is an ongoing controversy regarding the use of open or closed reduction methods for the surgical management of mandibular fractures. Proponents of open reduction claim that this technique has better nerve recovery and faster restoration of occlusal function than the closed reduction method [6]. In a systematic review by Andreasen et al., it was concluded that due to the heterogeneity and retrospective nature of all studies, definite conclusions cannot be drawn regarding the superiority of one surgical method over the other [7].

The current literature shows ambiguous results due to inconsistencies in treatment techniques, methods for the determination of neural sensation at baseline, variability in the follow-up period, and loss to follow-up [3]. Therefore, long-term follow-up studies are required to identify the risk factors for IAN recuperation and record IAN injuries in different types of mandibular fractures. The aim of the present study was to assess IAN recovery in various types of traumatic mandibular fractures treated with open reduction compared with the closed reduction surgical method, as no comprehensive study has been conducted on this topic.

## Materials and methods

Study design

This observational, prospective study was conducted in the Department of Oral Surgery, Jawahar Medical Foundation's Annasaheb Chudaman Patil Memorial (ACPM) Dental College, Dhule in association with Siksha 'O' Anusandhan Medical College from January 2022 to August 2023. The study was conducted following the Strengthening the Reporting of Observational Studies in Epidemiology (STROBE) guidelines for observational studies. Institutional ethical committee approval was obtained (EC/NEW/INST/2022/2959/2022/92) before starting the study and written informed consent was obtained from all patients after explaining the study to them, maintaining their confidentiality, and following the principles of the Declaration of Helsinki.

Sample size calculation

The sample size was calculated using G*Power software (version 3.1). A power analysis revealed that a sample size of 82 patients would provide a power of 80% (1-β), with an alpha value of 5% and an effect size of 0.44, as derived from a previous study [7]. Therefore, considering a 10% loss to follow-up, 90 patients were included in the present study.

Eligibility criteria

Patients aged > 18 years with unilateral mandibular fractures located between the lingual and symphysis due to road traffic accidents who were managed either with the open or closed reduction method, systemically healthy patients, patients with displacement of fracture segments < 5 mm, patients who were willing to participate, patients who could come for follow-up visits till six months, and patients with mental status to undergo preoperative neurosensory examination were included.

Patients who had associated head injuries or were unable to undergo preoperative neurosensory examination, edentulous patients, patients with bilateral mandibular fractures or fractures located proximal to the lingual such as condylar and coronoid fractures, comminuted fractures, fractures of the mandible managed conservatively, patients with associated pathologies of IAN, patients with a previous history of mandibular fractures, pathology, radiation therapy, extensive extra-oral incisions, and presence of other associated fractures such as zygomaticomaxillary and infraorbital fractures were excluded from the study.

Ninety patients, irrespective of sex, who were clinically and radiographically confirmed cases of unilateral mandibular fractures located between the lingual region and symphysis, were selected based on the eligibility criteria. They were divided into two groups using the non-probability purposive sampling technique: group 1 (45 cases) was treated with closed reduction, and group 2 (45 cases) was treated with open reduction and rigid internal fixation (RIF). All patients were treated within one to three days of reporting to the trauma center.

Method of neurosensory evaluation of inferior alveolar nerve

Demographic information was obtained for every patient, including age, sex, mode and cause of injury, time of injury, duration between sustained injury, and initial management or treatment. After obtaining consent from the patient and familiarizing the patient with the armamentarium used for neurosensory evaluation, four areas were selected: right and left lower lip and right and left chin area or mental nerve area. Testing for the IAN was performed over a 1 cm area above and beneath the labiomental fold of the injured and uninjured sides of the chin. The control side was the unaffected side of the patient’s face. Each of the four facial regions was subjected to three instances of stimulation. If the patient gave an accurate response for two of the three suitable responses, the sensation was normal. If accurate response was given only once, the patient had diminished sensation, and if no response was obtained, the sensation was absent. Throughout the examination, the participants were instructed to shut their eyes and position their lips in a relaxed manner.

CNT of IAN is commonly categorized into two fundamental groups, mechanoceptive and nociceptive, which are determined by the receptors that are activated through contact with the skin. Mechanoceptive testing can be further classified into two-point discrimination (TPD), static light touch (SLT), and brush directional stroke (BDS). In contrast, nociceptive testing can be subdivided into pinprick (PP) and thermal discrimination (TD). Nerve impairment was evaluated based on the grading algorithm proposed by Zuniga and Essick [4], who classified neurosensory testing into three levels: level A (TPD and BDS), level B (SLT), and level C (PP and TD).

Two-Point Discrimination Method

This test is designed to test for large, myelinated, slowly adapting A-alpha sensory nerve fibers. The test was performed using calipers with prongs set 5 mm apart and closed progressively until accurate responses were obtained according to Kawamura and Wessberg [8]. Campbell et al. reported that the normal measures for TPD in the trigeminal distribution vary from 7 to 14 mm; it is considered diminished at 15-20 mm and absent above 20 mm [9].

Static Light Touch and Brush Directional Stroke

These tests are used for large, myelinated, and quickly adapting A-alpha and A-beta nerve fibers. Von Frey monofilaments were used for SLT and testing started with 1.65 monofilaments, which was applied perpendicular to the skin until it just began to bend. In trigeminal nerve distribution, the detection of the 1.65 to 2.36 monofilament is generally considered normal for SLT [10]. In BDS, Von Frey monofilament was applied in a 1 cm stroke, three times in a zone from right to left and left to right. The patient was asked to accurately identify the direction on at least two out of three occasions.

Pinprick and Thermal Discrimination

These tests were used for A-delta and C fibers. The instrument utilized in conducting the pinprick examination entails a needle with a gauge of 22, which is grasped between the thumb and the index finger. It is administered under firm pressure in a rapid-pricking manner. The desired outcome of this procedure was to elicit a minor blood droplet at the puncture site, indicating that an adequate degree of force had been applied. An appropriate response should be feeling sharp, not dull, or experiencing intense pain. For the TD test, each facial region was gently treated with a cotton-tipped applicator containing ethyl chloride in addition to a substance with no therapeutic effect. The anticipated reaction was a sensation of coolness or coldness, and the patient was expected to be capable of distinguishing between placebo and ethyl chloride [10].

Scores corresponding to the level of neurosensory status were assigned as follows: score 0 as normal, score 1 as diminished, and score 2 as absent. The test was repeated thrice per individual by a single examiner trained in the neurosensory assessment, and the average of the three readings was considered during scoring. The neurosensation of the IAN was tested at baseline (preoperative) (T0), after one week (T1), three months (T2), and after six months (T3).

Surgical procedure

**Figure 1:**
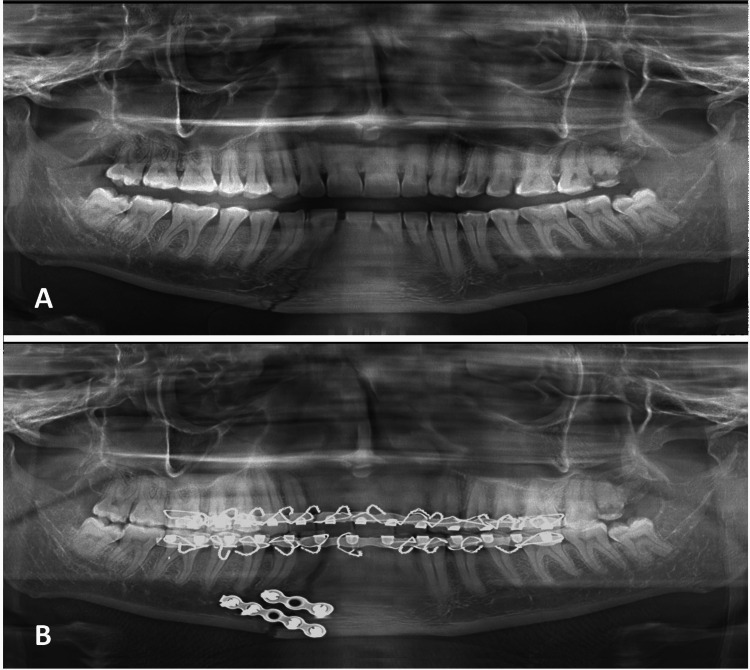
Orthopantomograph of mandibular body fracture treated by open reduction. (A) Pre-treatment. (B) Post-treatment.

For open reduction and RIF with 2-mm titanium mini plates and monocortical screws (6 mm), the plate was fixed to the fractured bony fragments (Figure 1).

**Figure 2:**
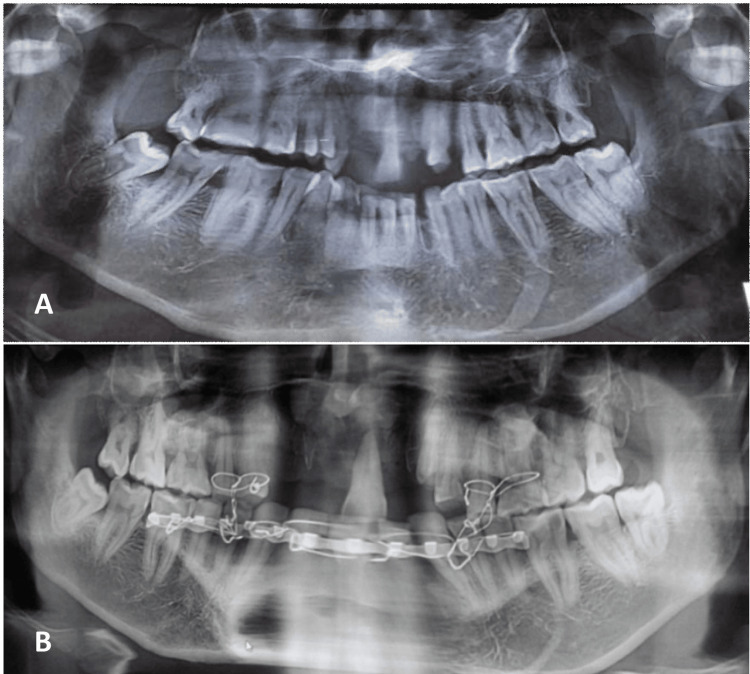
Orthopantomograph of mandibular parasymphysis fracture treated by closed reduction. (A) Pre-treatment. (B) Post-treatment.

Care was taken to place screws below or above the course of the IAN to avoid nerve injury. All patients underwent surgery under general anesthesia with nasotracheal intubation. Closed reduction and fixation were performed with rich arch-bar fixation under local anesthesia (Figure 2).

All patients underwent a postoperative orthopantomogram (OPG) to confirm the reduction and exclude any iatrogenic injuries (incorrect screw placement and damage to the inferior alveolar canal and mental foramen). None of the patients underwent maxillo-mandibular fixation after reduction. All surgeries were performed by experienced oral and maxillofacial surgeons.

Statistical analysis

Data were analyzed using SPSS version 22.0 (IBM Corp., Armonk, NY). The qualitative variables in the data, including gender and outcome variables (IAN recovery), are presented as frequency and percentage. The quantitative data, i.e., age, are presented as the mean with standard deviation. The chi-square test was used to assess the association of IAN recovery in both groups. The neurosensory scores for the different testing methods were compared using the Friedman test at pre- and postoperative time intervals. Statistical significance was set at P ≤ 0.05.

## Results

**Figure 3:**
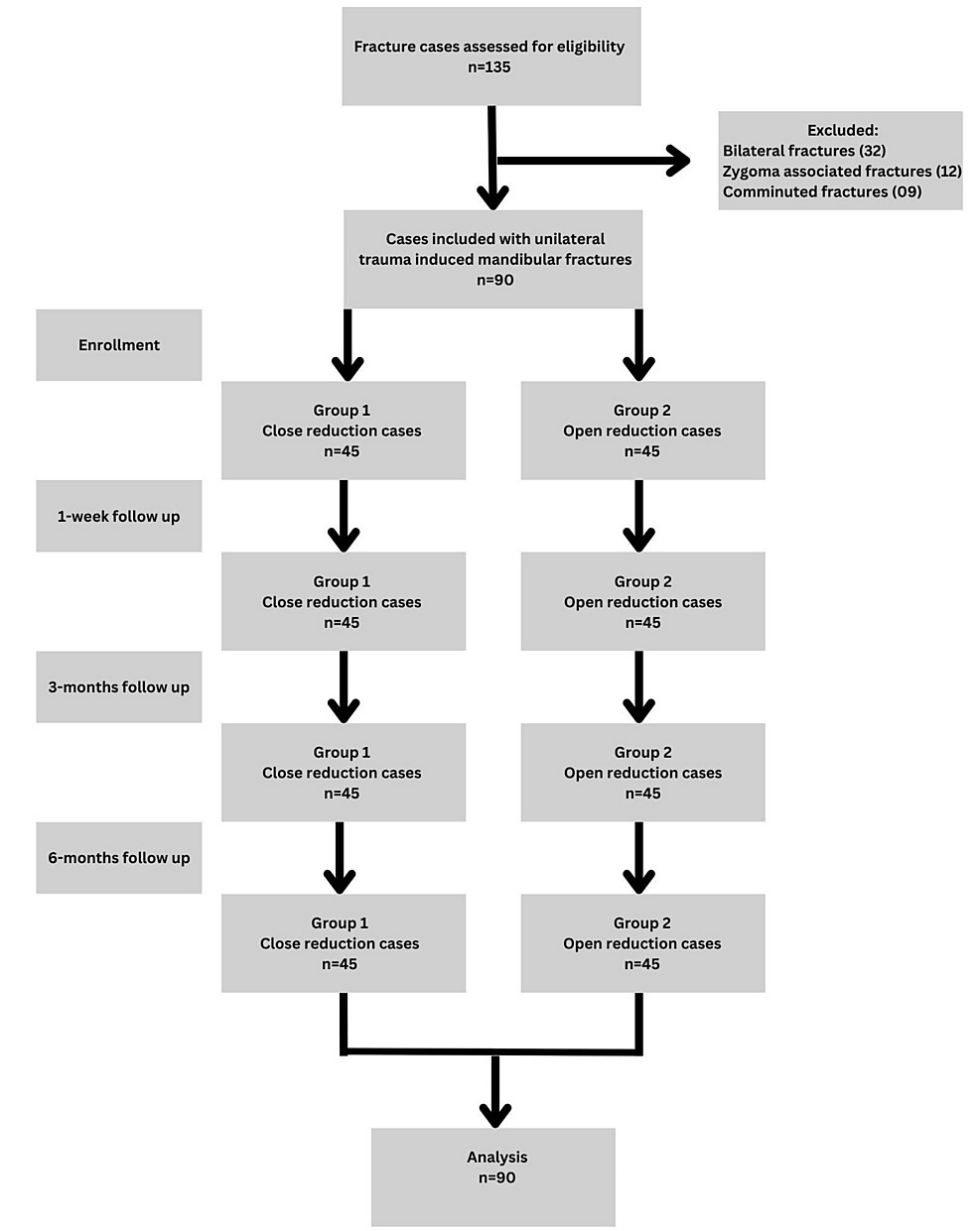
Strengthening the Reporting of Observational Studies in Epidemiology (STROBE) flowchart.

A total of 90 cases of unilateral traumatic mandibular fractures resulting from road traffic accidents were categorized into two distinct groups, each consisting of 45 cases. These groups were segregated based on the type of surgical treatment they received for fracture reduction, namely, open and closed reduction. All cases were examined objectively for neurosensory testing of IAN at baseline (preoperative), one week after surgery, three months, and at six-month follow-up, as shown in Figure 3.

Non-significant differences were noticed between the groups for baseline characteristics such as the mean age of the patients (34.2 ± 2.67 years in group 1 and 35.5 ± 3.68 years in group 2), sex (23 M/22 F in group 1 and 29 M/16 F in group 2), and site of injury. Fractures at the body of the mandible were the most common, followed by the angle and symphysis in both groups.

This eliminated the confounding bias due to age, sex, and site of injury. Functional nerve recovery was observed in 40 cases (88%) in group 1 and 38 cases (84%) in group 2, and the difference was not statistically significant (Table 1).

**Table 1:** Descriptive characteristics of the study groups. * Independent t test; ** chi-square test; NS: non-significant for p > 0.05.

Variables	Group 1	Group 2	p-value
Age (years)	34.2 ± 2.67	35.5 ± 3.68	0.061* (NS)
Gender			
Female, n (%)	22 (58%)	16 (42%)	0.205** (NS)
Male, n (%)	23 (44%)	29 (56%)	
Fracture sites			
Body, n (%)	16 (44%)	20 (56%)	0.573** (NS)
Angle, n (%)	15 (50%)	15 (50%)	
Symphysis, n (%)	14 (58%)	10 (42%)	
Functional nerve recovery			0.384** (NS)
Yes, n (%)	40 (88%)	38 (84%)	
No, n (%)	5 (12%)	7 (16%)	

**Figure 4:**
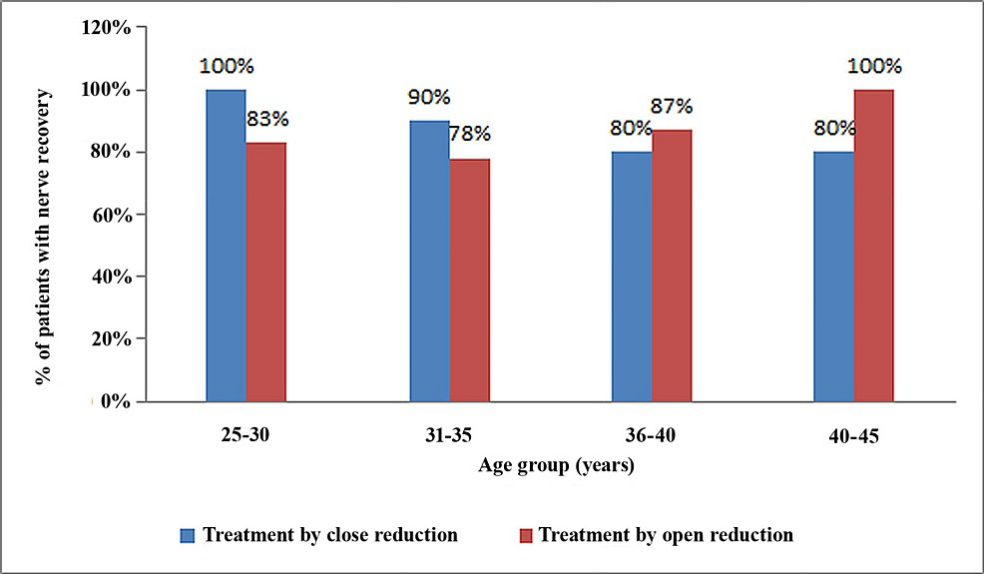
Percentage of recovered patients in both study groups according to age.

In the younger age group (25-30 years), both groups exhibited a more favorable recovery of IAN impairment. Conversely, as individuals grew older, diminished recovery was evident in both groups (Figure 4).

**Figure 5:**
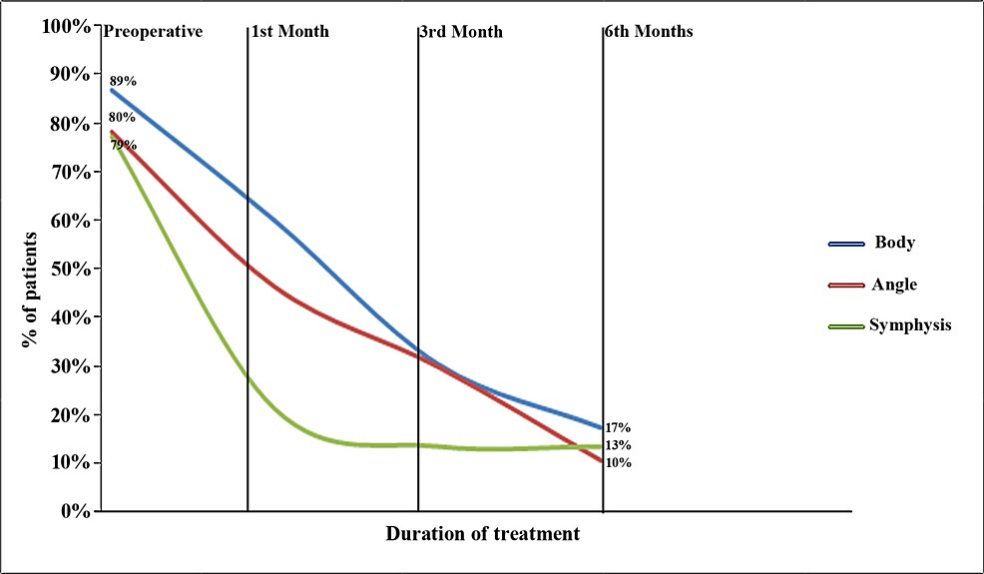
Percentage of patients with nerve dysfunction during the preoperative period and recovery during the follow-up period at different sites of injury.

During the preoperative period, a significant proportion of patients with body fractures (89%), angle fractures (83%), and symphysis fractures (36%) had nerve dysfunction. However, after a follow-up period of six months, the prevalence of nerve dysfunction decreased to 17% in body fracture cases, 14% in angle fracture cases, and 5% in symphysis fracture cases (Figure 5).

A total of 37 patients (82%) in group 1 and 38 patients (84%) in group 2 had abnormal nerve sensations during the preoperative period. After one week (postoperative period), 40 patients in group 1 and 40 in group 2 showed abnormal responses using the BDS method. A total of 39 patients in group 1 and 40 patients in group 2 showed an abnormal response to SLT. This showed an increased abnormal IAN response after surgery for level A and B testing, which was similar in both groups, whereas TPD and level C tests showed improvement after surgery in group 1 and no change in group 2. Both groups showed drastic improvement in CNT scores at all levels after three months and slow improvement thereafter until six months of follow-up. The total recovery in group 1 ranged from 60% to 80% and that in group 2 ranged from 56% to 72% (Table 2).

**Table 2:** The overall outcome of neurosensory status in study groups at different time intervals with different methods of clinical neurosensory testing (CNT). Level A tests: brush directional stroke (BDS) and two-point discrimination (TPD). Level B test: static light touch (SLT). Level C tests: pinprick (PP) and thermal discrimination (TD). T0: baseline; T1: one week after surgery; T2: after three months; T3: after six months.

Type of CNT	T0-T1		T1-T2		T2-T3		Total nerve recovery in Group 1	Total nerve recovery in Group 2	Average recovery in the follow-up period
	Group 1	Group 2	Group 1	Group 2	Group 1	Group 2			
BDS	-7%	-6%	60%	57%	13%	9%	66%	60%	63%
TPD	3%	1%	62%	62%	15%	11%	80%	72%	76%
SLT	-6%	-4%	63%	61%	12%	10%	69%	66%	68%
PP	1%	0%	58%	55%	8%	6%	67%	62%	65%
TD	1%	0%	52%	50%	7%	5%	60%	56%	58%

No significant differences were observed between the two groups in terms of neurosensory scores in patients with IAND at various time intervals. In group 1, 14 patients exhibited a decrease in sensory perception, while 23 patients displayed a complete absence of sensory perception of CNT. This number subsequently increased to 15 patients experiencing diminished sensation and 25 patients lacking any sensation after a period of one week after the operation. As time progressed, specifically after three months, the number of patients with diminished sensation decreased to only seven, and the number of patients with no sensation reduced to six. Further follow-up after six months revealed a further decrease in the number of patients with diminished sensation to three and a decrease in the number of patients without any sensation to four. In group 2, 17 patients exhibited a decrease in sensory perception, while 21 patients displayed a complete absence of sensory perception of CNT. This number subsequently increased to 19 patients experiencing diminished sensation and 22 patients lacking any sensation after a period of one week after the operation. As time progressed, specifically after three months, the number of patients with diminished sensation decreased to only five, and the number of patients with no sensation reduced to four. Further follow-up after six months revealed a further decrease in the number of patients with diminished sensation to two and a decrease in the number of patients without any sensation to three (Table 3).

**Table 3:** Comparison of inferior alveolar nerve scores in patients between both groups at different time intervals. T0: preoperative; T1: one week postoperative; T2: three months; T3: six months; NS: non-significant for p > 0.05.

Time interval	Groups	N	Normal	%	Diminished	%	Absent	%	P-value
T0	Group 1	45	8	18%	14	31%	23	51%	0.799 (NS)
	Group 2	45	7	16%	17	38%	21	47%	
T1	Group 1	45	5	11%	15	33%	25	56%	0.699 (NS)
	Group 2	45	4	9%	19	42%	22	49%	
T2	Group 1	45	32	71%	7	16%	6	13%	0.616 (NS)
	Group 2	45	36	80%	5	11%	4	9%	
T3	Group 1	45	38	84%	3	7%	4	9%	0.821 (NS)
	Group 2	45	40	89%	2	4%	3	7%	

No statistically significant disparities were observed in CNT scores during the preoperative phase, indicating that both groups exhibited comparable modifications in the neurosensation of the IAN. However, group 2 demonstrated significantly higher scores in TPD, SLT, and TD testing at T1 (one week postoperative) and PP testing at T2 (three months follow-up). This suggests that the open reduction method may have affected the neurosensation of the A-alpha, A-delta, and C fibers, which subsequently recovered at the six-month follow-up period (Table 4).

**Table 4:** Friedman test analysis for multiple neurosensory test methods at different time intervals. BDS: brush directional stroke; TPD: two-point discrimination; SLT: static light touch; PP: pinprick; TD: thermal discrimination; T0: preoperative; T1: one week postoperative; T2: three months; T3: six months; * p < 0.05: significant; NS: non-significant for p > 0.05.

Time intervals	TO		T1		T2		T3	
	Group 1	Group 2	Group 1	Group 2	Group 1	Group 2	Group 1	Group 2
BDS	8	8.5	6.5	6.5	3.5	3.5	1.5	2
TPD	7.5	8	6	6.5	3	3	2	1.5
SLT	7.5	7.5	5.5	6	3.5	3.5	1.5	1.5
PP	8.5	8.5	6	6	3	4	1	0.5
TD	7.5	8	6	6.5	3	3	0.5	0.5
P-value	0.862 (NS)		0.002*		0.041*		0.391 (NS)	

## Discussion

Traumatic mandibular fractures have a high incidence and are among the most common fractures of the face [1]. The choice between open and closed reduction for traumatic mandibular fractures remains controversial. Historically, these fractures have been exclusively treated using closed reduction owing to a variety of factors, including complications related to the IAN, technical difficulties, and the potential for facial scarring following surgical intervention. Typically, closed reduction fracture techniques involve less pain and fewer complications than surgical options. Still, closed reduction carries some risks such as the surrounding nerves, blood vessels, and soft tissues may become damaged and the bone may not heal properly as a result of improper alignment. The open reduction methods allow direct visibility and accessibility to reduce bony fragments; however, they can lead to an increased risk of infection, swelling, and mobility of bony plates [5-7,9]. Notably, satisfactory outcomes have been achieved by using a conservative approach. Damage to the IAN can occur due to an accident or a surgical procedure. The occurrence of post-traumatic or pre-treatment dysfunction of the IAN following a fracture of the mandible varies between 5.7% and 58.5%, whereas the occurrence of IAND following treatment varies between 0.9% and 66.7% [2]. The vast array of prevalence estimates provides evidence for the absence of consistency in both documented occurrences and methodologies employed to evaluate neurosensory impairment [3]. Therefore, the objective of the present study was to compare IAN recovery with open reduction and internal fixation with closed reduction for the management of traumatic mandibular fractures.

There are various confounding factors, such as age, sex, site of injury, duration between trauma and surgical procedure, displacement of bony fragments, and time of immediate postoperative examination. No statistically significant differences were observed in the mean age of the patients, sex distribution, or site of injury, which reduces bias due to these factors. However, nerve recovery was better in the younger age group and decreased with age. Aging affects the functional and electrophysiological characteristics of the peripheral nervous system. This includes a reduction in the velocity at which nerves conduct signals as well as a decline in muscle strength, sensory discrimination, autonomic responses, and blood flow within the endoneurium [11].

In the present study, males had more traumatic mandibular fractures than females in both groups. This could be due to the inclusion of cases of mandibular fractures caused by road traffic accidents. Fractures of the body and angle were more common than those at the symphysis and were associated with neurosensory loss in the IAN. This finding is in agreement with previous studies [12]. A plausible explanation for the higher occurrence of IAND in fractures of the mandibular angle and body can be attributed to the exposure of the IAN. Normally protected within the mandibular canal, IAN becomes vulnerable when fractures occur in the angle or body region. In these cases, the fracture line is likely to traverse the mandibular canal, resulting in tension and compression forces acting on the IAN [13].

In our study, the duration of treatment after trauma was zero to three days. According to Tabrizi et al., the shorter the span between trauma and time of surgical management, the better will be the recovery of IAN [14]. In our study, the displacement of the bony fragments was < 5 mm. It has been postulated that displacement of segments > 5 mm significantly increases the risk of IAND [15]. Postoperative examination of all patients was performed one week after the surgical procedure. Patients who have undergone a closed reduction procedure with the use of local anesthesia may not undergo an examination on the day of surgical intervention because of the enduring effects of local anesthesia. Furthermore, the initial postoperative neurosensory assessment may be postponed for up to seven days following treatment [15].

Postoperative nerve disturbances may occur because of trauma or fracture management. Managing a mandibular fracture can be achieved through either open reduction internal fixation (ORIF) or closed reduction. Neurosensory disruptions were predominant in individuals who underwent open reduction. The nerve can experience trauma after compression, dissection, or stretching by surgical instruments or bone fragments [11]. In the present study, no significant differences were observed between the open and closed reduction methods. This is in contrast with previous studies [6,15]. This could be due to the presence of confounding factors, such as lag time between injury and treatment, displacement of bony fragments, and time of postoperative evaluation. In our investigation, monocortical screws were used exclusively in all the participants. Furthermore, following the surgical procedure, OPGs indicated the absence of screw encroachment in the mandibular canal. Consequently, the occurrence of neurosensory impairment due to screw encroachment was negative. It was also hypothesized that this could be due to the inclusion of a specific group of patients with fractures with minimal displacement. These patients had fewer occurrences of functional impairment when initially seeking medical attention.

In the present study, it was noticed that CNT scores improved drastically after three months, and then slowly improved till six months. The changes from the first postoperative day to one week postoperatively were minimal. A total of 60-80% of cases recovered their neurosensation after six months of follow-up, which was similar to previous studies [12]. Different studies have documented different recovery rates [12], which could potentially be accounted for by the varying approaches utilized to evaluate the functionality of the IAN, as posited by the study conducted by Poort et al. in 2009 [13].

Nerve regeneration exhibits a rapid pace within the initial three months, and subsequently decelerates the rate of recovery. The duration of recuperation, contingent on the gravity of the trauma, may span several months. Sufficient periodic evaluation is important for monitoring recovery from neurosensory disturbances. One week is insufficient time for any major changes to occur. Vriens et al. selected a minimum interval of three months because of the anticipation that most regenerative histologic reactions to trigeminal nerve injury would have recovered within this timeframe [16]. Donoff posited that if a nerve has not spontaneously recovered within six months, it is unlikely to do so [17]. When the IAN undergoes severance or impairment, it initiates an intrinsic reparative process. The nerve fibers, commonly known as axons, undergo retraction and enter a phase of dormancy lasting approximately one month; subsequently, they commence regeneration. The axons exhibit regrowth at a rate of roughly 1 mm per day and recover within three to four months. This time may vary depending on the severity of nerve injury [18]. Therefore, in our investigation, we opted to utilize follow-up intervals of three and six months to assess any improvement in sensory function and evaluate any remaining paresthesia.

It was also observed in our study that level A and B responses were affected more than level C responses, and there was a statistically significant difference in the responses after the surgical procedure. This could be due to the fact that the A-δ fibers, which are myelinated but have a smaller diameter, along with the C fibers, which are unmyelinated, have the responsibility of nociception, which includes pain and temperature perception. Owing to their more primitive nature, unmyelinated fibers are less susceptible to compression-related injuries than larger-diameter A-δ fibers, which are myelinated and involved in mechanoreception. Consequently, after experiencing a compression-induced injury, it is common to observe unaffected sensations of pain and temperature discrimination but impaired perception of light and moving touch. The reaction to thermal differentiation exhibited remarkable performance, and it was the primary capability that was restored [19,20]. Blomqvist et al. endorsed this notion of swift advancement in the restoration of thermal stimuli as a result of the accelerated regeneration of A-delta small myelinated sensory fibers. The examination of thermal differentiation may yield affirmative results, even in cases where all other assessments indicate diminished sensitivity [21].

In the current study, postoperative two-dimensional OPG was used to assess the impingement of monocortical screws on the IAN. Three-dimensional cone beam computed tomography scans should be performed to better evaluate pre- and postoperatively. McDonald et al. proposed the use of magnetoencephalography as an objective method to assess post-traumatic IAN injuries. This examination aids in distinguishing between intact and impaired nerves and between severed nerves [22]. Previous studies have postulated that the average measurement from the external buccal cortex to the inferior alveolar canal in the mandible of individuals is 6.97 mm, with a minimum measurement of 4.8 mm. It has been observed that the implementation of monocortical screws exceeding 6 mm in length can potentially result in injury to the nerves located within this particular anatomical region [23]. In our study, to avoid IAND, monocortical screws with a diameter of 6 mm were used.

Limitations of the study

Our research methodology has certain constraints, as the treatment groups were not selected randomly, leading to an elevated potential for spurious associations resulting from selection bias. There was also an absence of pre- and postoperative three-dimensional imaging to assess the extent of nerve damage and to ascertain whether the plating screws exerted pressure on the IAN, as this was not considered a customary course of action. However, the assessment of the impact of plate fixation on IAN injury was not the primary aim of this investigation. Only objective methods of CNT were used; however, tingling and numbness can be better evaluated using subjective methods. Long-term follow-up of more than six months was not performed in this study; therefore, future prospective studies should be conducted with long-term follow-up.

## Conclusions

IAN recovery after traumatic mandibular fracture was not statistically different between open and closed reduction procedures. Based on the findings of the present study, the authors recommend both methods for surgical management of traumatic fractures of the mandible, taking other factors into consideration. Recovery of the nerve was observed in 60%-80% of the cases. Nevertheless, the neurosensory condition of the IAN deteriorated following treatment, especially in relation to the clinical neurosensory testing conducted at levels A and B. However, significant improvement was observed at the three-month follow-up, followed by gradual enhancement at the six-month follow-up.
